# Target-flanker similarity alters the spatial profile of visual crowding

**DOI:** 10.1167/jov.25.12.17

**Published:** 2025-10-08

**Authors:** Kristian P. Skoczek, Jennifer H. Acton, John A. Greenwood, Tony Redmond

**Affiliations:** 1 School of Optometry and Vision Sciences, Cardiff University, Cardiff, UK; 2 Experimental Psychology, UCL, London, UK

**Keywords:** crowding, psychophysics, spatial vision

## Abstract

Visual crowding is the disruptive effect of nearby details on the perception of a target. This influence is dependent on both spatial separation and perceived similarity between target and flanker elements. However, it is not clear how these simultaneous influences combine to produce the final “crowded” percept as flankers traverse the limits of the crowding zone. We investigated the reported appearance of a peripherally presented Landolt-C target flanked by a pair of simultaneously presented Landolt-Cs across different levels of target-flanker similarity (relative orientation), spatial separation, and target eccentricity. The distributions of errors in reported target orientation were fitted with a pooling model that simulated errors using a weighted combination of target and flanker orientation signals. The change in error distribution with target-flanker spacing (the “spatial profile”) was fitted with a logistic function, estimating both the rate at which target- and flanker-signal weighting varies as target-flanker spatial separation decreases (slope) and the spatial separation at which signals were balanced (midpoint). We found that the slope of the spatial profile increases as target-flanker similarity decreases, with similar modulation patterns across target eccentricities. In contrast, spatial profile midpoints increased linearly with eccentricity, in line with Bouma's law, but were invariant of target-flanker similarity. This suggests similarity-related modulation may operate within a fixed spatial extent at each eccentricity. Investigating the spatial profile of crowding disentangles effects related to the appearance of targets and flankers (i.e., similarity) from appearance-independent influences, which can be confounded when using other common measures to define crowding zone extent.

## Introduction

Crowding is the disruptive effect of nearby distractors on the perception of object details. These distractors disrupt object perception within a restricted spatial extent (Bouma, 1970), which we refer to here as the “crowding zone.” Presentation of other objects or details within the “crowding zone” around a target leads to a deterioration in identification (but not detection) of target features (Pelli, Palomares, & Majaj, 2004). Measures of crowding zone extent frequently rely on estimates in the form of either “threshold-spacing”; the target-flanker spacing necessary to achieve a set threshold level of performance at a crowding task, or “critical spacing” (Pelli et al., 2004), the maximum target-flanker spacing necessary to elicit a change in task performance from an uncrowded level.

Crowding is known to be a cortical phenomenon (Flom, Heath, & Takahashi, 1963), although the exact mechanisms remain debated. A number of proposals link the observed extent of crowding interactions to cortical properties such as the distance between target and flankers on the cortical surface (Levi, Klein, & Aitsebaomo, 1985; Pelli, 2008), cortical receptive field size (He, Wang, & Fang, 2019; Motter, 2009) and receptive field overlap (Dayan & Solomon, 2010; Greenwood, Jerotic, Danter, Finnie, & Schwarzkopf, 2023; van den Berg, Roerdink, & Cornelissen, 2010). The link between these anatomical properties and psychophysical estimates of the spatial extent of crowding zones is complicated by observations that crowding effects are influenced not only by target-flanker separation, but also the perceived similarity between target and flanking stimuli (Estes, 1982; Kooi, Toet, Tripathy, & Levi, 1994). Elevations in threshold and critical spacing have been interpreted as an indicator of larger crowding zones when estimated with particular stimuli (Kooi et al., 1994; Shamsi, Liu, & Kwon, 2022). Alternatively, it has been suggested that similarity effects may arise from differences in “amplitude” of the effects of crowding within a fixed spatial extent at a particular visual field location (Pelli & Tillman, 2008).

Visual crowding induces systematic, as opposed to random, changes to target appearance (Parkes, Lund, Angelucci, Solomon, & Morgan, 2001), which typically follow the perceived appearance of the flanker features (Bernard & Chung, 2011; Greenwood, Bex, & Dakin, 2010; Harrison & Bex, 2015). These predictable changes in appearance can be well described by pooling models which suggest that the perception of a crowded target arises from a weighted combination of target and flanker signals (Greenwood, Bex, & Dakin, 2009; Harrison & Bex, 2015; Parkes et al., 2001). Methods allowing observers to directly report perceived details of a crowded target (Agaoglu & Chung, 2016; Ester, Klee, & Awh, 2014; Ester, Zilber, & Serences, 2015; Harrison & Bex, 2015; Kalpadakis-Smith, Tailor, Dahlmann-Noor, & Greenwood, 2022) allow these integrative mechanisms to be more directly targeted than would be possible with other “forced-choice”-style methods. These methods may also help to avoid spurious interpretation arising from limited selections of response options (Reuther & Chakravarthi, 2020). Harrison and Bex (2017) showed that the relative proportion of response types varies with target-flanker spacing, and that relative similarity between stimulus elements also influences the kinds of errors that are made (Harrison & Bex, 2015). However, it remains to be investigated how these simultaneous influences of target-flanker similarity and spacing interact to give the final perception of a crowded target as flankers traverse the limits of the crowding zone. Investigating this interaction will also reveal whether the dimensions of the crowding zone change in the presence of similar vs dissimilar flankers, or if observed differences arise from modulations of the magnitude of crowding effects occurring within a constant zone extent.

Here, we examine the simultaneous influences of target-flanker separation and target-flanker similarity on perceived target appearance. By exploring how target perception is affected by flanker appearance at a range of target-flanker separations spanning the limits of the crowding zone, we aim to characterise how target-flanker similarity may impact estimates of crowding zone extent achieved with other popular methods. We present the “spatial profile of visual crowding”—a model describing the distributions of errors in reported target appearance when flankers are presented at varying target-flanker separations within the crowding zone. Analogous to a psychometric function of crowding with respect to spatial separation, the model reveals novel indicators of crowding mechanisms which disentangle factors contributing to the magnitude and spatial extent of crowding. We also present spatial profiles of crowding assessed at three levels of target-eccentricity to assess how the similarity-based modulation of crowding effects differs within the mid-peripheral visual field.

## Methods

To examine the influence of target-flanker similarity on the appearance of a crowded target, the distributions of errors in reported target orientation were compared in the presence of flankers at different relative orientations and target-flanker spacings. Participants reported the perceived orientation of a randomly-oriented Landolt-C target under crowded and uncrowded conditions. In crowding trials, the target was flanked by a pair of Landolt-C flankers, spaced along the tangential axis and identical to each other in orientation (offset from the orientation of the target). Ethical approval for the study was gained from the Cardiff University School of Optometry and Vision Sciences Ethics Committee (Ref. no.: 1507, February 5, 2020), and all experiments conformed to the Declaration of Helsinki.

### Participants

Six psychophysically trained participants (authors, one female, two male, and three males unaware of the study objectives) were recruited to take part in the study. All participants gave informed consent and underwent initial screening tests to ensure normal ocular and visual health. Refractive correction was required for two participants, who wore spectacles with single-vision lenses during the experiments. Participants wore an eye patch over their left eye throughout data collection.

### Experimental setup

Stimuli were presented on a gamma-corrected OLED display (Sony PVM-A250 Trimaster EL; Sony Corp., Tokyo, Japan), calibrated with a ColorCal MKII photometer (Cambridge Research Systems, Kent, UK). A viewing distance of 57cm was used when testing at 12.7° target eccentricity, and a distance of 40cm was used when testing at 4.2° and 21.2° target eccentricity. Stimulus generation and presentation was conducted with MATLAB (version R2019a; The MathWorks Inc., Natick, MA, USA) using PsychToolbox (Brainard, 1997) and the Eccentric Vision Toolbox (Greenwood, 2021). A rotating volume dial was used by participants to rotate a presented response Landolt-C along a continuous scale and register its perceived orientation after each stimulus presentation. Room lights were switched off and participants were adapted to the flat gray background (10 cd · m^−^^2^ luminance) for two minutes before beginning and after any breaks between blocks of trials. Exported data were analysed with R (version 4.1.2, 2021-11-01) (R Core Team, 2021) with the “circular” package (Agostinelli & Lund, 2022).

### Experimental task

**Figure 1.**
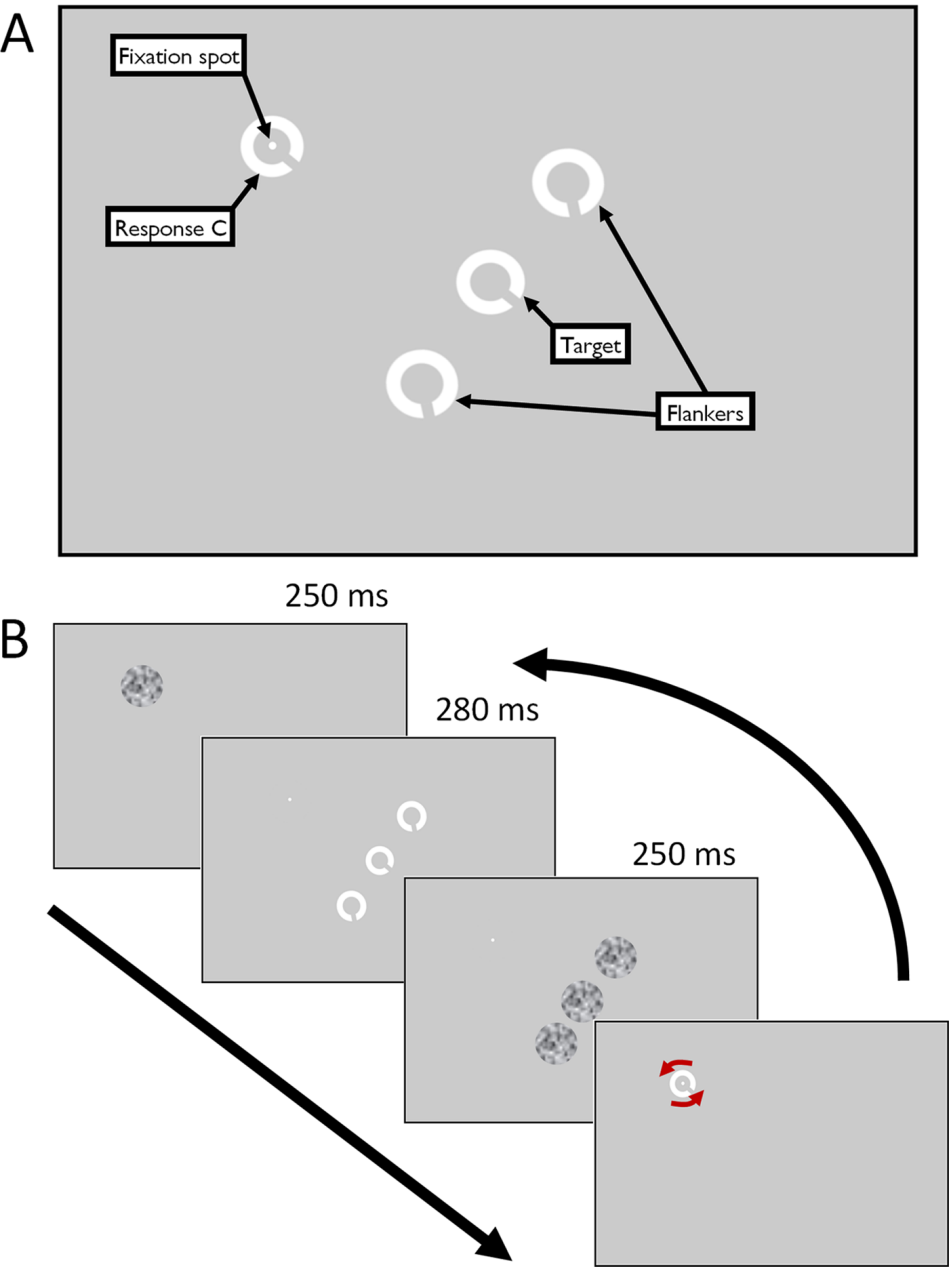
(**A**) The main screen elements that appeared at various times through a trial (not to scale). (**B**) The timeline of a typical crowded trial. The response C was presented at a random orientation and rotated by the participant with a response dial (indicated by curved red arrows that were not presented during trial). After matching the perceived orientation of the target, the next trial began.

In each trial, participants fixated a Gaussian spot target (approx. 0.2° diameter) with a target Landolt-C presented below and right of fixation (inferotemporal), to the right eye of each participant. To induce crowding, two additional flanker Landolt-Cs were presented at a fixed separation from the center of the target C along the axis tangential to the axis of eccentricity (see Figure 1). Four levels of target-flanker orientation difference were sampled at each target eccentricity, randomly interleaved within blocks of trials. Unflanked trials were also interleaved among flanked trials. Flankers were both oriented (identically to each other) at one of 4 possible orientations relative to the target: 22.5°, 45°, 90°, and 180°, randomly clockwise or anticlockwise. All stimuli (targets and flankers) were presented for 280 ms, after which a 250 ms random noise mask was presented at the locations of the target and flankers to disrupt after-images (each presented in a gaussian window of diameter equal to twice the stimulus diameter). After each presentation and noise mask, a response Landolt-C was presented at a random orientation, centered at the fixation spot. Participants controlled the orientation of the response C with the dial, rotating it to their perceived orientation of the target, and submitted their response with a button press. After a response was given, a random noise mask was presented centered at the fixation spot for 250 ms, followed by only the fixation spot for 500 ms, before the next trial began. Three repeats of the full test procedure were conducted per participant. Participants were encouraged to be as accurate and precise as possible without rushing, and to take breaks when needed.

Trials were presented in distinct blocks at each eccentricity (4.24°, 12.72°, and 21.2°) so that the locations of the fixation spot and target Landolt-C were constant within each block. Data were collected in two rounds, first at 12.72° eccentricity for 150 trials per condition, then interleaved blocks at 4.24° and 21.2° for 80 trials per condition (the investigation initially involved one eccentricity, and was later expanded to include the two other eccentricites for further investigation). “Condition” here refers to each combination of target-flanker orientation difference, spatial separation (including unflanked) and target eccentricity, giving 3150 trials at 12.72° eccentricity and 1680 trials at 4.24° and 21.2° eccentricity in total. Flanker appearance (relative orientation difference, target-flanker spacing and unflanked condition) was shuffled and presented in a randomized order within each block (save for the 45° orientation difference at the smallest and greatest eccentricities, which was added later in a separate session in order to expand the investigation and aid interpretation of initial findings). Stimulus diameter (for the target and flankers) was scaled for eccentricity at each location following Equation 1, below.

(1) $$\begin{array}{c} Diameter=\frac{\left(E+1.77\right)*1.5}{12.72+1.77}\; \end{array}$$

Here, E = target eccentricity in degrees of visual angle to equate stimulus size to a target of 1.5° diameter at 12.72° eccentricity. This equation was adapted from Horton & Hoyt (1991) and uses parameters obtained from fitting their equation to fMRI data collected from a healthy cohort of similarly-aged controls (Wright, 2021). The calculated target diameters were 0.6° for targets at 4.24° eccentricity, and 2.4° in diameter when the target was at 21.2° eccentricity.

### Calculating response error

Response errors were calculated as the difference between the target orientation presented at each trial and the response orientation. The sign of errors made in flanked trials was transformed so that, relative to the target orientation, errors toward the orientation of the flanker were positive, and errors away from the orientation of the flanker were negative. For example, if flankers on a given trial were oriented clockwise relative to the target, a response 10° clockwise relative to the target orientation would give an error of 10°, whereas a response of 10° anticlockwise would give an error of −10°, and vice versa in the case of anticlockwise flankers. The probability of each possible error (−175° to 180° in 5° steps) being reported was calculated by summing the number of trials producing each error and dividing by the total number of trials conducted for each unique combination of target-flanker spacing and relative orientation difference.

### Model-fitting to distributions of response errors

Unflanked data were fit with a single von Mises distribution centered at 0° with two free parameters, corresponding to the mean orientation and spread (or concentration, *k*). For each flanked condition (target-flanker orientation difference and spacing), the probabilities of potential errors (the frequency of each error divided by the total number of trials) under each condition were then fitted with a model comprised of a weighted mixture of three von Mises distributions: two distributions of variable concentration (8 ≤ *k* ≤ unflanked concentration), one of which was centered at 0° ± 10°, the second fixed to the value of target-flanker orientation difference, and the third a uniform distribution (*k* = 0, no true mean) to capture random errors (Equation 2). The model therefore contains 5 free parameters: weighting factors for target-centered and random error distributions, concentration parameters for target-centered and flanker-centered distributions, and the mean orientation of the target-centered distribution (0° ± 10°). Model parameters were fit by minimising the sum of squared errors between observed probabilities of report errors and the model.

(2) $$\begin{array}{ccc} & & \;P_{\left(\theta \right)}=w_{R}*f\left(\theta \;|\;0,0\right)+\left(1-w_{R}\right)*((w_{T}*f\left(\theta \;|\;\theta_{T},k_{T}\right)\;\\ & & \;+\;\left(1-w_{T}\right)*f\left(\theta \;|\;\theta_{F},k_{F}\right)) \end{array}$$

f() = *von Mises distribution*
θ = *Response error*
W = *Free weighting factors (Random, Target)*
θ_T_ = *Mean of target-centered distribution (0° ± 10°)*
θ_F_ = *Mean of flanker-centered distribution (fixed to target-flanker orientation difference)*
k = *Free concentration parameters (Target, Flanker)*

Modelling responses as this mixture of distributions permits calculation of the relative proportions of the responses made under different conditions by comparing weighting parameters for each of the “target” and “flanker” centered response distributions. The proportion of errors centered around 0° was taken as the probability of giving a response centered at the orientation of the target, abbreviated to p(Target). A value of 1 would indicate that responses were centered around the target orientation, whereas a proportion of 0 would indicate all responses were centered around the flanker orientation. As the model allows the von Mises distributions associated with target- and flanker-centered responses to overlap and add together, individual trials cannot be designated as being “target-centered” or “flanker-centered.” Responses occurring between the orientations of the target and flankers on a particular trial can be captured through the mixture of the two distributions and their respective weightings. This approach is similar to pooling models that simulate the distributions of neural responses to the target and flanker elements (Harrison & Bex, 2015; Kalpadakis-Smith et al., 2022), although we focus more on the response distributions to more directly examine the contribution of target- and flanker-centered response errors.

### Fitting spatial profiles of crowding

To quantify the effect of target-flanker spacing on the distributions of response errors, the change in p(Target) values across the range of target-flanker spacings tested was modelled with a logistic function with two free parameters—location and gradient (Equation 3). This describes the spatial profile of crowding associated with each target-flanker orientation difference. The location parameter (midpoint) of this function indicates the target-flanker separation at which non-random responses switch from mostly target-centered to mostly flanker-centered. The gradient of the logistic function is steepest at the midpoint and determines the span of the transition zone between minimal crowding (fewer flanker-centered responses) and maximum crowding (fewer target-centered responses).

(3) $$\begin{array}{c} p\left(Target\right)=\;\frac{1}{1+e^{\left(\frac{\;S-L\;}{G}\right)}}\; \end{array}$$

S = *Target-flanker spatial separation*
L = *Location*
G = *Gradient*

Spatial profile parameters were fitted with a nonlinear least squares model to minimise the sum of squared errors between observed p(Target) values and those predicted by the model. Data were weighted by 1/SSE of each p(Target) value so that values derived from pooling models that better reflect the underlying distribution of errors were weighted more heavily than those from poorer fitting models. Additionally, the midpoint parameter was limited to a maximum value equal to the target eccentricity to aid model fitting. The span of the transition zone was estimated by predicting the target-flanker spacing associated with p(Target) values of 0.4 and 0.6 (indicating 40% and 60% of responses centered at the target orientation respectively) and calculating the difference in spacing between them. This meant shallower spatial profiles of crowding equated to a wider span of the transitionary zone.

The effects of target-flanker orientation difference and target eccentricity on the spatial profile of crowding (specifically the midpoint and span) were each tested with linear mixed effects models analysis, conducted in R (version 4.1.2, 2021-11-01) (R Core Team, 2021). The model included participant as a random effect on the intercept, and fixed effects of eccentricity and target-flanker orientation difference. Model comparison was conducted with analysis of variance (ANOVA) against a “null” model with only the fixed effect of eccentricity and participant as a random effect on intercepts, to investigate the presence of an effect of target-flanker orientation difference. As the trends in values of the span of the spatial profile were visibly non-linear, log transformation was used to linearize the trend in the data for these analyses.

An error affecting one repeat of the procedure was noted after data collection for participants 1-3. During this run at 12.7° target eccentricity, the smallest target-flanker separation was incorrectly set to a value larger than the intended 1.5° visual angle. The incorrect separation was different for each participant, but all were between 1.5° and 2.0° (1.6°, 1.7° and, 2.0°). The remaining two repeats of the experiment were unaffected.

## Results

**Figure 2.**
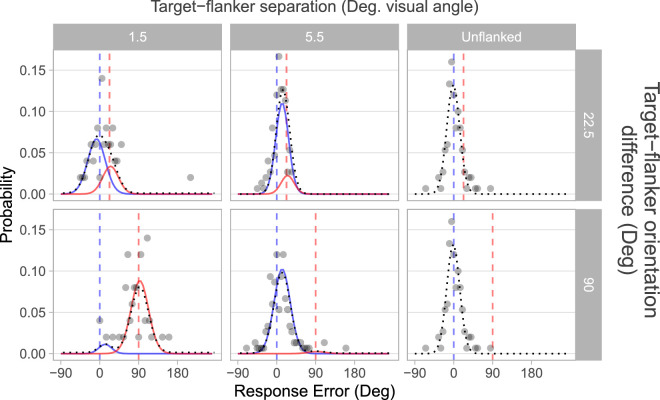
Example distributions of response errors and model fitting. Distributions of response errors from participant 3 at 12.7° eccentricity, at low (left) and high (middle) target-flanker spatial separations and orientation difference (rows). Unflanked errors are shown in the right column, with the same data in both rows for ease of comparison to flanked data. Values of target-flanker separation (degrees of visual angle) and orientation difference (degrees of orientation) are given in gray labels. The dotted lines show the fitted mixed von Mises model, solid lines show the underlying target- and flanker-centered distributions (blue and red, respectively). Vertical dashed lines indicate the orientation of targets (blue), and flankers (red). Where target- and flanker-centered distributions overlap (upper row), the peak of the mixed distribution occurs between the target and flanker orientations. When present, the orientation of the flankers (both clockwise and anti-clockwise to the target) corresponds to the positive value of the target-flanker orientation difference, as the sign of errors in the presence of anti-clockwise flankers were flipped. Because the data are circular (i.e., orientations on a continuous and repeating scale), the x-axis of these distributions wrap from −90° to 270° for optimum presentation of data.

**Figure 3.**
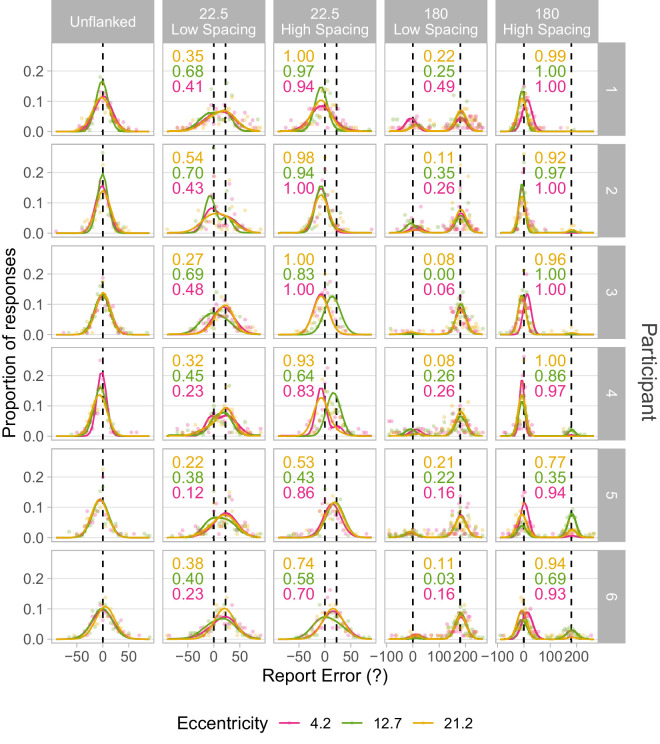
Example distributions of response errors from all participants and eccentricities. Data points indicate the proportion of responses made at each error away from target orientation (0, vertical dashed line). Unflanked responses are shown in column 1. Columns 2 and 3 show data observed with flankers at 22.5° offset from the target (also indicated by vertical dashed line) at the smallest (Low Spacing) and largest (High Spacing) target-flanker spatial separation examined at each eccentricity. Columns 4 and 5 show data observed with flankers at 180° orientation offset from the target. Solid lines show pooling models fitted to the data at each eccentricity, indicated by color. Fitted values of p(Target), the proportion of target-centered responses, are shown numerically on each panel.

**Figure 4.**
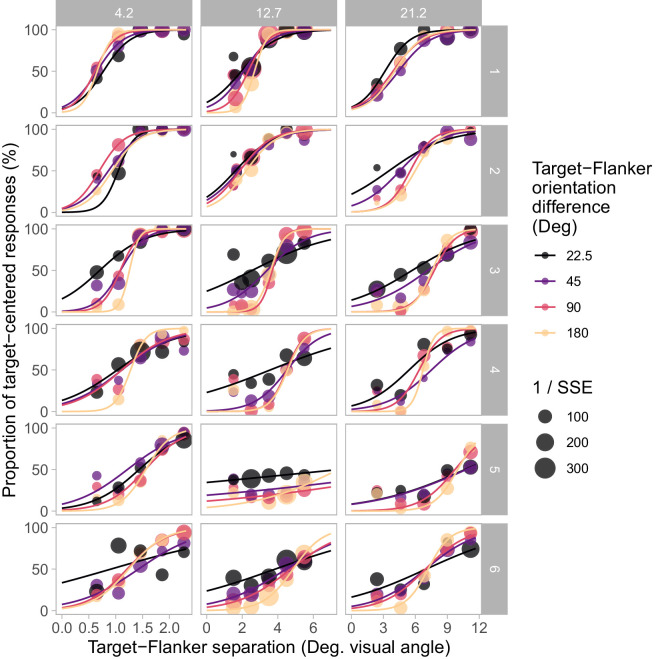
Spatial profiles of crowding by target-flanker orientation difference and target eccentricity. Data points indicate the proportion of non-random responses centered at the orientation of the target (y-axis) derived from models fitted to errors in reported target orientation. The proportion of target-centered responses increases with target-flanker spatial separation (x-axis). The size of data points indicates the goodness of fit of the underlying pooling model fitted to response error distributions, quantified as the inverse of the sum of squared errors (1/SSE). Data for each participant are presented within each row, and data for different levels of target eccentricity are separated across columns. Solid lines indicate fitted spatial profile models, with associated target-flanker orientation difference indicated by color. Greater target-flanker orientation difference (pink and yellow data) was associated with steeper spatial profiles than smaller orientation difference (purple and black lines).

The presence of flankers induced systematic errors in the reported orientation of the target which were well described by the fitted pooling models (examples plotted in Figures 2 and 3). The change in target appearance due to the presence of flankers at varying distances is characterised by the spatial profile of crowding, plotted in Figure 4, which describes the increase in the proportion of target-centered signal in responses as target-flanker spacing is widened. Target-flanker similarity is found to influence the range of spacings over which this change in response distribution occurs (the span of the “transition zone”), whereas the center of this transition (the midpoint) is unaffected (Figure 6). This similarity-based modulation of spatial profiles is proportionally similar over the range of target-eccentricities tested (Figure 7), whereas the midpoint proportionally increases with eccentricity. Although common methods of estimating crowding zone extent may confound crowding magnitude and spatial extent, the spatial profile of crowding provides independent indicators of these factors.

### Distributions of errors

Response errors in the unflanked condition were concentrated around an error of 0°, corresponding to the presented orientation of the target. Fitted von Mises distributions described the data well, and indicated participants were able to report the target orientation with reasonable reliability (fitted parameters are given in Supplementary Marterials). The greatest mean error (corresponding to the peak of the distribution) for these probability distributions was −1.6°, suggesting there was no systematic bias in responses in the unflanked trials.

Responses in all conditions where flankers were present (particularly with smaller target-flanker spatial separations) tended to cluster around the target orientation and the flanker orientation in varying proportions. When the target-flanker difference in orientation was greater, crowded responses followed a bimodal distribution as shown in the data for the 90° orientation difference in Figure 2. When the target-flanker orientation difference was smaller, a unimodal distribution was found, with a peak in error orientations between those of the target and flanker (see data for the 22.5° orientation difference in Figure 2). With the greatest target-flanker spacings (which should induce weaker or absent crowding), errors were typically unimodally distributed around 0, though sometimes with a slightly wider spread in responses compared to the unflanked condition, as can be seen in Figure 3.

The proportion of responses centered around the target, referred to as p(Target), was consistently greater with increasing target-flanker separation. When flankers were presented adjacent to the target such that they were just touching, responses tended to be mostly centered at or around the flanker orientation. Flanker-centered responses particularly dominated at small spacings between targets and flankers with greater orientation difference (90° or 180°). Conversely, a proportion of responses remained close to the target orientation when neighboring flankers had smaller orientation offset from the target (22.5° and 45°). Best fitting parameters for all fitted pooling models are given in the Supplementary Materials (note that the weighting of flanker-centered responses is calculated as 1 – target-centered weighting, so these values are not shown).

### Spatial profiles of crowding

The proportion of responses centered at the target orientation, p(Target), were plotted against spatial separation for each participant and each target-flanker orientation difference (Figure 4). In general, p(Target) increases as target-flanker spatial separation widens. The data show a systematically steeper shift in p(Target) with increasing target-flanker orientation difference. For large target-orientation differences, this change in performance occurs over a shorter range of target-flanker spatial separation. Conversely, smaller target-flanker orientation differences are associated with a more gradual change in the distribution of responses as target-flanker spacing is widened. Best fitting parameters describing each spatial profile of crowding are given in the Supplementary Materials.

**Figure 5.**
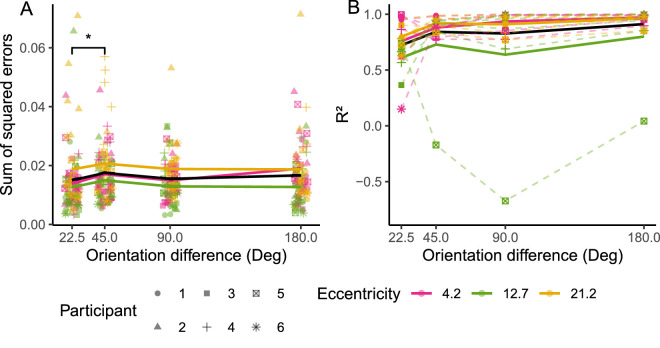
Goodness-of-fit for pooling models (**A**) and spatial profile models (**B**). (**A**) The sum of squared errors (SSE) associated with each fitted pooling model (note: low SSE is indicative of a good model fit). Asterisks indicate a statistically significant difference between levels of target-flanker orientation difference, identified with Tukey's HSD (*p < 0.05). (**B**) The calculated *R*^²^ value associated with fitted spatial profile models against target-flanker orientation difference. *R*^²^ is indicative of the proportion of variance in the underlying p(Target) data that is explained by the model for each dataset. In both panels, bold lines indicate the mean of the data at each level of target-flanker orientation difference and for each level of target eccentricity (indicated by color). The proportion of variance that can be explained by the model is generally high for the majority of fitted spatial profiles.

The reliability of the fitted spatial profile models was assessed by examining the sum of squared errors of fitted pooling models and the coefficient of determination (*R*²) of fitted spatial profiles, shown in Figure 5. The sum of squared errors of pooling models (Figure 5A) were consistently low across levels of target-flanker orientation difference and target eccentricity, though ANOVA of log-transformed SSE values found main effects of eccentricity (*F*(2, 360) = 16.48, *p* < 0.001) and target-flanker orientation difference (*F*(3,360) = 2.99, *p* = 0.031). Post hoc Tukey's HSD showed the 12.7 eccentricity SSE values were statistically significantly lower than those from the other eccentricities, but no statistically significant difference was found between 4.2 and 21.2 eccentricity data. The Tukey test also found SSE values corresponding to 45° target-flanker orientation difference to be statistically significantly higher than those of 22.5°, but no statistically significant differences were found between other levels of orientation difference (importantly: no difference in SSE was found between 180° and 22.5° orientation difference data). Overall, this indicates that there was no systematic influence of either orientation difference or eccentricity on the goodness-of-fit of the pooling models, and therefore that the p(Target) values (the proportion of target-centered responses) used in the fitting of spatial profile models were reliable. Data at 12.7 eccentricity were collected earlier than data from the other eccentricities, and with a greater number of trials per condition (150 at 12.7, 80 at both 4.2 and 21.2), so it is unlikely that this finding could be due to a potential learning effect lingering after the practise trials. Rather, the additional trials are the likely origin of elevated goodness of fit (indicated by lower SSE values) at this eccentricity.

The spatial profiles that were then fit to these p(Target) values can be assessed by calculating *R*^2^ as 1 – (sum of squares Error/sum of squares Total), which compares the errors between the data and the fitted model (sum of squares Error) with the variation of data around the mean (sum of squares Total). The majority of spatial profiles were associated with an *R*² of over 0.7, indicating that at least 70% of the variance in p(Target) values can be explained by these models. *R*² values were high with variations in both eccentricity and target-flanker orientation difference, though tended to be lower with the smallest orientation difference. There were however 5 spatial profiles associated with *R*^2^ values of less than 0.5, with three of these arising from one participant at one eccentricity. Two of these values (from participant 5, 12.7°) were negative, which arises from the above calculations of *R*^2^, indicating that the fitted model describes the data more poorly than a straight line fitted through the mean of the data (i.e., driven by the flat, noisy functions for this participant). Because these poor fits only appeared at one eccentricity, the participant's data was retained in later statistical analyses. Nonetheless, on average the spatial profile models explain more than 83% of the variance in p(Target) values. *R*^2^ values with participant 5’s data removed are presented in Supplementary Figure S2 for improved clarity.

**Figure 6.**
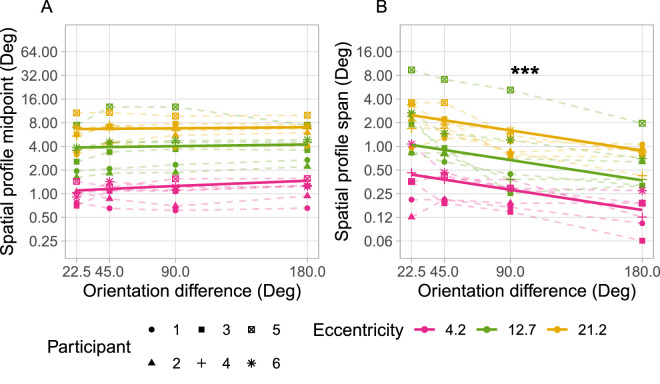
Midpoint (**A**) and span (**B**) of fitted spatial profiles of crowding for each participant (dashed lines). Solid lines show main effects of fitted linear mixed models. The asterisks indicate a significant effect of target-flanker orientation difference on spatial profile span indicated by the likelihood ratio test of mixed effects models (*p* < 0.001). Target-flanker orientation difference modulated the span of spatial profiles of crowding but did not significantly influence the target-flanker spacing at which responses are equally distributed around target and flanker orientations. Further comparisons with significant differences are described in the text but are omitted here for clarity.

We quantified the spatial profile of these variations in two ways, beginning with the midpoint of the spatial profiles—the spatial separation at which performance is exactly midway between veridical and maximally crowded responses (Figure 6A). Fixed effect parameters and 95% confidence intervals estimated by the linear mixed effects model were: eccentricity = 0.326 [0.279, 0.374] and orientation difference = 0.002 [−0.003, 0.008]. These results indicate that the spatial profile midpoint data is unaffected by target-flanker similarity in terms of relative orientation. This is further supported by a likelihood ratio test of the linear mixed model to the “null” model (fixed effect of eccentricity and random intercepts by participant only), which did not indicate that the effect of orientation difference was significant (*p* = 0.403).

We also examined the slope of the spatial profile of crowding via the span of the transition zone; the difference in target-flanker spacing corresponding to p(Target) values of 0.4 and 0.6. This value is greatest where target-flanker orientation difference is smallest (Figure 6B). Span estimates are smaller at larger orientation differences because the spatial profile of crowding is steeper (more “step-like”), as seen in Figure 4, indicating the shift in responses occurs over a smaller range of target-flanker separations. The span of spatial profiles was also generally wider (spatial profiles were shallower) at greater eccentricities. Fixed effect parameters and 95% confidence intervals were: eccentricity = 0.148 [0.121, 0.174] and orientation difference = −0.010 [−0.013, −0.006]. The likelihood ratio test of the model against the “null” model indicated that the effect of orientation difference on spatial profile span is significant (*p* < 0.001). A second likelihood ratio test compared the mixed effects model with a model including an interaction between effects of eccentricity and target-flanker orientation difference (with scaled and centered values of eccentricity and orientation difference). No statistically significant interaction between the effects was indicated (*p* = 0.654). This suggests that the change in spatial profile span with orientation difference was similar at each eccentricity tested.

**Figure 7.**
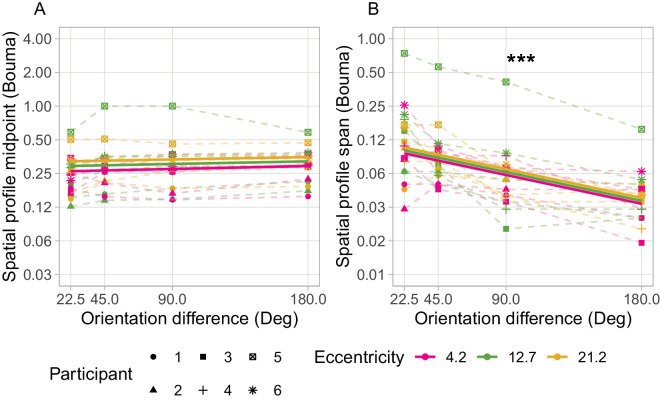
Midpoint (**A**) and span (**B**) of fitted spatial profiles converted to a ratio of spatial distance to target eccentricity (Bouma units). Solid lines show the main effects of linear mixed effects models. Target eccentricity is represented by the color of lines and data points. The asterisks indicate the significant effect of target-flanker orientation difference on spatial profile span indicated by the likelihood ratio test of mixed effects models (*p* < 0.001). The midpoint of spatial profiles was similar across eccentricities and target-flanker orientation differences when expressed as Bouma units. The span of spatial profiles was also similar across eccentricities when expressed as Bouma units, but significant differences between values of span assessed with different target-flanker orientation differences remain. The similarity-based modulation of the span of the spatial profile of crowding is similar across the range of target eccentricities investigated.

Both the midpoint extent and span of the spatial profiles of crowding increase with target eccentricity. This echoes earlier findings that crowding zone extent increases approximately linearly with eccentricity (Bouma, 1970); a finding that as been reported repeatedly (Pelli & Tillman, 2008) and termed “Bouma's law.” This linear relationship predicts that transforming the spatial data to “Bouma-units,” by dividing target-flanker spacing by the target eccentricity, would collapse the data across levels of target eccentricity. As seen in Figure 7, converting to Bouma units greatly reduced the disparity between values of midpoint and span seen across eccentricities, and almost aligns the mean values. Estimated fixed effect parameters and 95% confidence intervals for midpoint data were: eccentricity = 0.003 [0.000, 0.007] and orientation difference = 0.000 [−0.000, 0.001]. The effect of target-flanker orientation difference on midpoint was again, not statistically significant (*p* = 0.343). A second likelihood ratio test compared the “null” model (fixed effect of eccentricity and random intercepts by participant), with a further reduced model of random intercepts only (no factor for eccentricity). This test suggested that the effect of eccentricity on spatial profile midpoint expressed in Bouma units was still statistically significant (*p* = 0.045). However, the parameter estimate was very small (3.48 × 10^−3^), and the lower confidence interval was also close to zero (9.99 × 10^−^^5^).

The span of the spatial profile in Bouma-units is consistent across target eccentricities for each level of target-flanker orientation difference (see Figure 7). Estimated fixed effect parameters and 95% confidence intervals were: eccentricity = 0.010 [−0.013, 0.034] and orientation difference = −0.010 [−0.012, −0.007]. The effect of target-flanker orientation difference on span was again statistically significant (*p* < 0.001). A second likelihood ratio test compared the model with another alternative “null” model with a fixed effect of orientation offset and random intercepts by participant. This test suggested that the effect of eccentricity on spatial profile span expressed in Bouma units was not statistically significant (*p* = 0.386). This indicates the similarity-based modulation of the spatial profile is constant across eccentricity when expressed as a ratio of the target eccentricity (i.e., Bouma-units). Although the parameter estimates for the effect of orientation difference and eccentricity are similar, note that the confidence intervals around the orientation difference parameter are much tighter and not inclusive of zero.

As mentioned above, the data for participant 5 at 12.7 degrees eccentricty were retained in these statistical analyses despite notably poor indicators of model goodness of fit. When the statistical analyses were repeated with these data removed, all statistically significant effects remained, main effect parameters were similar and confidence intervals were narrower (shown in Supplementary Figure S1). This suggests that the inclusion of the poorly fitting data did not introduce any effects that would otherwise not be indicated.

## Discussion

We set out to examine the effect of target-flanker similarity on the spatial profile of crowding using continuous estimates of target appearance and a model-fitting approach. Our findings suggest that target-flanker similarity modulates, at least in part, the span of the transitionary zone between minimum and maximum crowding effects, with no notable effect on the overall position of the spatial profile of crowding. Greater target-flanker orientation difference (reduced similarity) leads to a sharper transition between “fully crowded” and “no crowding” distributions of responses, compared to smaller orientation differences. Increasing target eccentricity has a dual effect of shifting the midpoint of the spatial profile of crowding further from the target and producing generally shallower spatial profiles (thus giving a wider transitionary span). However, spatial profiles of crowding appear similar between eccentricities when target-flanker spacing is expressed as a ratio of target eccentricity (Bouma-units), indicating that similarity-based modulation of the transitionary span, and similarity-independence of the midpoint, is preserved between the target eccentricities.

### Distributions of response errors

Varying target-flanker spacing altered the relative contribution of target- and flanker-orientation signals in the resulting distribution of errors in reported target orientation. This echoes the findings of other researchers using similar paradigms which allow participants to report target appearance on a continuous scale (Harrison & Bex, 2015; Harrison & Bex, 2017; Põder, 2012). The shift in contribution is well captured by the relative weighting of two von Mises distributions, centered at the target- and flanker- orientations, which are then combined similarly to previous population pooling approaches (Greenwood & Parsons, 2020; Harrison & Bex, 2015; Kalpadakis-Smith et al., 2022).

Earlier studies comparing distributions of responses at two levels of target-flanker spatial separation (Ester et al., 2014; Ester et al., 2015) report greater proportions of errors around the flanker orientation at smaller separations, in agreement with other studies reporting spacing-dependent modulation of crowding strength (Kooi et al., 1994; Pelli et al., 2004; Tripathy & Levi, 1994). However, it is difficult to determine whether these data agree with the findings presented here (of similarity-based modulation in transitionary span) due to the combining of data across the full range of target-flanker orientation differences (Harrison & Bex, 2015; Harrison & Bex, 2017), or the instantiation of a “trimodal” pooling model to describe the distributions of errors observed (Ester et al., 2015). It is consequently difficult to differentiate the effects of target-flanker orientation difference from that of target-flanker separation in much of the data presented in those studies. Nevertheless, one report (Ester et al., 2015) does indicate a higher proportion of flanker-centered errors with flanker orientation offset by 60° relative to the target, compared to those offset by 15°, at the smallest spatial separation between target and flankers, while probabilities were equally low at the larger spatial separation. This agrees with the findings of the data presented here: that greater target-flanker orientation differences lead to proportionally fewer target-centered responses at target-flanker separations smaller than the midpoint of the spatial profile.

### The spatial profile of crowding

The finding that the specific weighting of target- and flanker-related signals is dependent on both target-flanker spacing and similarity has been noted in recent literature (Greenwood & Parsons, 2020; Kalpadakis-Smith et al., 2022). Fitting the two-parameter model to the crowding data revealed that target-flanker similarity has little influence on the midpoint of the spatial profile. Instead, the midpoint is primarily driven by the target eccentricity. The finding that similarity-dependent aspects of the spatial profile of crowding (the transitionary span) and relatively similarity-independent aspects (location of the midpoint) can be differentiated in psychophysical data presents a new approach to investigating the edge and extent of the crowding zone.

The negligible effect on the midpoint of the spatial profile across different levels of target-flanker similarity provides an insight into the potential low-level anatomical structures that could contribute to the spatial extent of the crowding zone. Relevant structures include cortical receptive fields (in terms of their extent (He et al., 2019; Motter, 2009) or overlap (Dayan & Solomon, 2010; Greenwood et al., 2023; van den Berg et al., 2010), which in turn may have spatial properties inherited from ganglion cell receptive field density across the retina (Kwon & Liu, 2019). It is of course difficult to find a direct correspondence between psychophysical measures of crowding and underlying neural properties (e.g., from retinotopic analyses). The results of the present study suggest that estimates of crowding zone extent that are sensitive to manipulations of flanker appearance have the potential to weaken any association with neural data. Although it is beyond the scope of this article to directly link these properties with behavioral measures, our findings present a novel indicator of crowding zone extent that may be relevant to future work. We hypothesize that the midpoint of the spatial profile could provide an indirect marker of a speculative “outer limit” of possible crowding interactions beyond the psychophysically observable extent, that is also robust against effects of target-flanker similarity. The extent of this outer limit, which would also be relatively invariant of target-flanker similarity, could represent the maximum reach of the receptive fields that provide any input to the crowding mechanism. The similarity-dependent weighting of responses to target and flanker features would then occur within this hypothetical outer limit at each target location. Flankers located close to this outer limit may contribute to the internal population response (Kalpadakis-Smith et al., 2022; van den Berg et al., 2010) but be weighted so low as to be undetectable by typical psychophysical techniques, such as forced-choice responses. The midpoint of the spatial profile may then serve as an indicator of this similarity-independent outer limit, which would increase proportionally with eccentricity (following the observed changes in the midpoint in Figure 6) matching an identifying feature of crowding (Bouma, 1970; Pelli et al., 2004).

### Implications for current and future research

**Figure 8.**
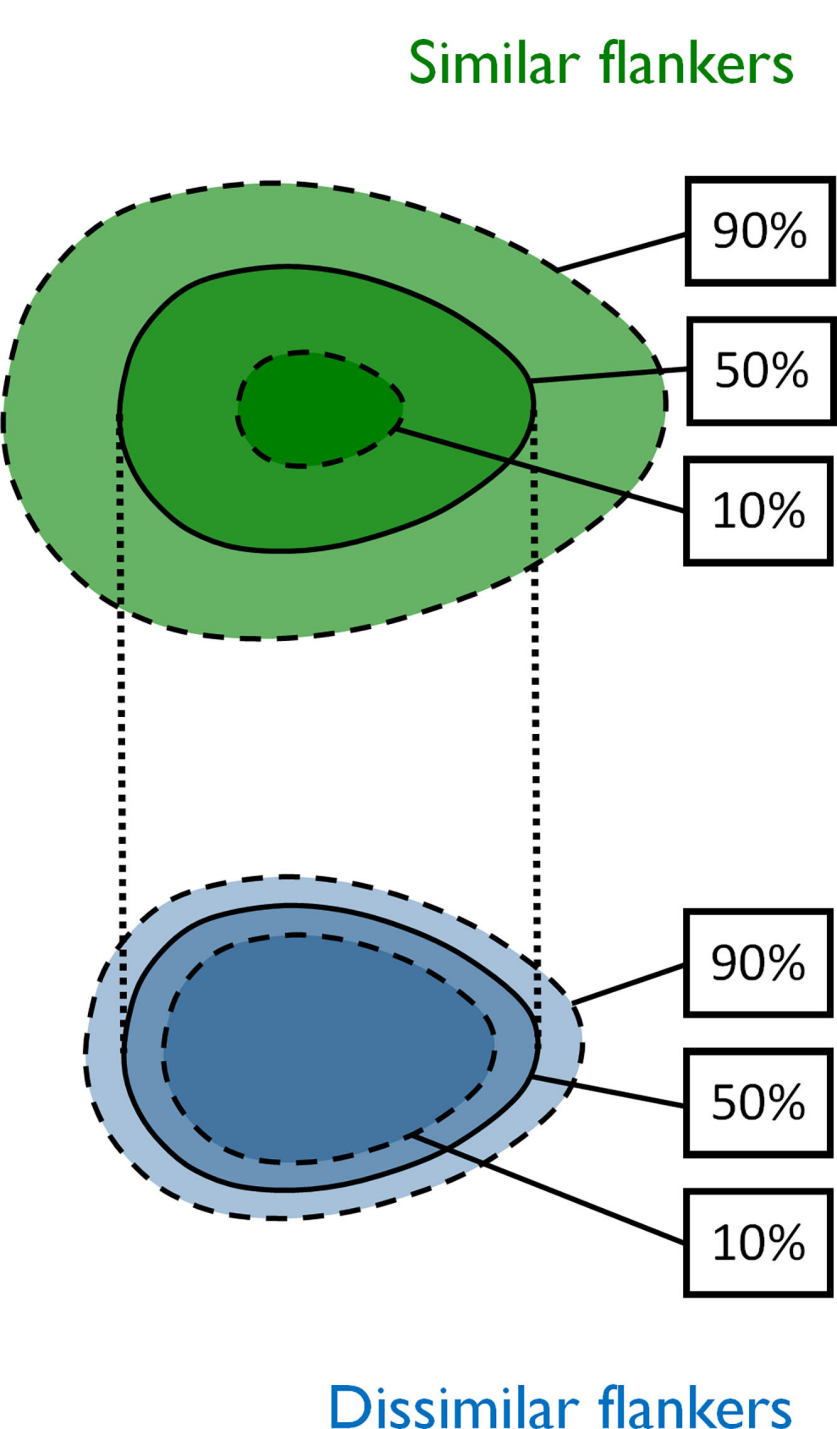
Schematic illustration of the spatial profile of crowding and how a change in the transition zone (rather than midpoint spacing) may lead to an apparent shift crowding zone extent in threshold- or critical spacing-based measurements. Upper and lower shapes illustrate crowding zones associated with similar (green) and dissimilar (blue) target-flanker appearance by extrapolating samples of the target-flanker separation indicated by hypothetical spatial profiles that correspond to 90%, 50% and 10% target-centered responses. Dotted lines indicate the shared midpoint-spacing, while dashed lines highlight that different performance criteria produce varying measures of crowding zone extent. Crowding zones may appear larger when flankers are more similar in appearance to the target, but the shared midpoint may suggest a common physiological origin of the extent of spatial interactions.

These findings also have important implications for experimental design in studies of crowding, particularly when interpreting and comparing crowding studies. Wider transitionary spans between minimum and maximum crowding under certain experimental conditions (as seen with greater target-flanker similarity), or in different clinical conditions, could well give the impression of a larger extent of the crowding zone when estimated with a criterion-level performance above 50%. A schematic illustration of this effect can be viewed in Figure 8. Such criteria are widely used in the literature, for instance in comparing typical observers with clinical disorders of vision (Greenwood et al., 2012; Ogata et al., 2019). It is therefore unclear whether these reported elevations in crowding may be explained by increased spatial extent, an altered transitionary span, or a combination of both types of changes. It is difficult to disentangle the relative contribution of these plausible changes or to adequately infer one type of change over another when using only a single criterion measure.

Although not observed here, either independent or concurrent shifts in midpoint and span of the spatial profile may also be possible under different experimental conditions. Both the location of the midpoint and the span of the spatial profile of crowding could theoretically contribute to findings of differences in criterion-based estimates of the extent of the crowding zone such as threshold- or critical-spacing. This means that a single threshold- or critical spacing-based measure alone is unable to identify how the underlying spatial profile may be altered due to experimental manipulations or between individuals. Because either the midpoint or span of the spatial profile, or both, may be indicative of different features of crowding, establishing which one (or combination) of these is altered under different experimental conditions may lead to stronger associations with other functional or structural measures and a clearer understanding of the effects of physiological or pathological processes. This may be particularly relevant in studies examining visual crowding in the presence of pathology (Greenwood et al., 2012; Ogata et al., 2019) where underlying physiological structures related to crowding zone extent may be expected to be abnormal. It is currently uncertain whether reports of altered crowding zone extent with visual pathology are the result of changes in the extent of the “outer limit” of crowding interactions, or dysfunctional weighting of target and flanker signals within an unchanged spatial extent. Each possibility may have profoundly different implications for understanding the pathophysiology of these conditions.

As noted above, some fitted spatial profile models describe the underlying p(Target) data better than others. There is also some noise apparent in the parameter estimates obtained. These factors warrant some amount of caution in interpretation of the data. Although absence of a statistically significant effect is not itself definitive evidence that there is no such effect, the stark difference in trends of the fitted parameters in the presented data (between panels A and B in both Figures 6 and 7) strongly suggests that while the spatial profile span changes with target-flanker orientation differences, the midpoint appears largely invariant. Alternative analyses carried out with ANOVA of the fitted spatial profile parameters also gave the same outcomes, validating that the choice of statistical test did not influence the final conclusions drawn.

In the current study, we have taken the orientation difference between target and flanker Landolt-Cs as an intuitive and quantifiable measure of target-flanker similarity. A full consideration of the variation in crowding with target-flanker similarity should also include extreme values, such as identical target and flanker orientations (0° difference), which have recently been found to give improved performance in some instances (Cicchini, D'Errico, & Burr, 2022) though not in others (Ozkirli, Pascucci, & Herzog, 2025). In our paradigm this condition would be difficult to model—any “flanker-centered” responses induced by crowding from an identical flanker would also be centered at the same orientation as the target, thus giving a p(Target) value of 1 at all target-flanker spatial separations. A similar assessment of the spatial profile of crowding made with a different measure of crowding magnitude, would not be expected to be incompatible with the findings presented here. We hypothesize that the midpoint of the spatial profile of crowding is indicative of a fundamental anatomic origin of the extent of crowding zones, and there is no reason to expect identical flankers to present a special case in this regard.

## Conclusions

Our results suggest that the spatial profile of crowding can be differentially influenced by target-flanker appearance and eccentricity. The finding that the midpoint varies with target eccentricity (and not notably with target-flanker similarity) suggests that the spatial extent of crowding at a particular visual field location is constant, with variations in crowding strength at the outer limits of the crowding zone that may bias estimates of zone extent. The critical spacing approach to measure crowding (and other criterion-based measures greater than 50% performance) may conflate changes in the midpoint and span of the spatial profile of crowding, weakening associations with other functional and structural measures along the visual pathway. Such commonly used psychophysical methods for measuring crowding may also overlook potentially informative changes in the underlying spatial profile. It also cannot be ruled out that weak (or absent) associations between crowding zone “extent” and other functional or structural measures in previous literature may in fact be the result of using psychophysical methods that target portions of the spatial profile of crowding that do not precisely describe the true extent of the crowding zone.

Target-flanker similarity was found to modulate the span of the spatial profile of crowding, and the pattern of this modulation was similar at all levels of eccentricity investigated. Therefore the spatially-dependent weighting of responses to flanker features in weighted pooling models is different for flankers that are similar in appearance to the target, than for those that appear dissimilar. Differential weighting of responses to flankers that are similar and dissimilar to the target has been indicated in earlier studies (Greenwood & Parsons, 2020; Kalpadakis-Smith et al., 2022) and could potentially be the origin of similarity effects noted elsewhere (Ester et al., 2015). The findings presented in this paper add that the modulation of weightings according to orientation difference may be predictable across target-flanker spacings within the crowding zone, and that this pattern of modulation is similar across the levels of eccentricity investigated.

## Supplementary Material

Supplement 1
