# Supplementary material for: Target-flanker similarity alters the spatial profile of visual crowding
Source: J Vis. 2025 Oct 8;25(12):17. doi: 10.1167/jov.25.12.17 (PMC12517358; doi:10.1167/jov.25.12.17)
Supplement: Supplement 1 [file jovi-25-12-17_s001.docx]

# **Supplementary Materials**

Tables of fitted model parameters are available in a repository hosted on the Open Science Framework at the following link: <https://osf.io/ey48u/?view_only=4db15fac0c13450cbe5d62d9039e30b1>

# **Supplementary figures**

**Figure S1** – *Midpoint (A) and span (B) of fitted spatial profiles of crowding for each participant (dashed lines, participant 5, 12.7 eccentricty data removed). Likelihood ratio tests again indicated no statistically significant effect of orientation difference on midpoint (p = 0.96), and a statistically significant effect on span (p = <0.001, indicated by asterisks). The main effect of target-flanker orientation difference on spatial profile span persists, and in comparison with the full data set presented in Figure 6, estimated parameters are similar but 95% confidence intervals were slightly narrower: eccentricity = 0.148 [0.128, 0.167], orientation difference = -0.010 [-0.12, -0.07].*

**Figure S2** *- Calculated R² value associated with fitted spatial profile models against target-flanker orientation difference, with participant 5’s data removed. R² is indicative of the proportion of variance in the underlying p(Target) data that is explained by the model for each dataset. Bold lines indicate the mean of the data at each level of target-flanker orientation difference and for each level of target eccentricity (indicated by colour). The proportion of variance that can be explained by the model is generally high for the majority of fitted spatial profiles.*
